# In this month’s Bulletin

**DOI:** 10.2471/BLT.22.001122

**Published:** 2022-11-01

This month’s theme issue is rehabilitation. In editorials, Director-General Tedros Adhanom Ghebreyesus (654) explains how the World Health Organization supports its member states in the provision of rehabilitation services. Alarcos Cieza et al. (655) make the case for change in the way these services are organized, staffed and funded. 

Fid Thompson (658–659) reports on how Ukraine is building on rehabilitation work initiated by the government before the war. Abderrazak Hajjioui talks to Tatum Anderson (660–661) about the need to prioritize multidisciplinary rehabilitation education, practice and research.

## India

### Reaching rural populations 

Rajani Mullerpatan et al. (662–668) pilot sustainable services in five villages.

## Israel 

### Access to audiologists 

Limor Lavie & Tali Bar-Moshe (739–743) examine lessons learnt after increased subsidies for hearing aids.

## Italy

### Physiotherapy in primary care 

Alessandra Da Ros et al. (669–675) track referrals to a family and community physiotherapist.

## Japan 

### Three large databases 

Kaori Yamaguchi et al. (699–708) explore uses of insurance data in managing rehabilitation services.

## South Africa

### What is provided in primary care? 

Thandi Conradie et al. (689–698) compare national treatment guidelines.

## Viet Nam

### Improving physiotherapists’ training

Didier Demey et al. (733–738) link four Vietnamese universities with international providers of pre-service education.

## Global

### How to best manage post COVID-19 condition

Simon Décary et al. (676–688) review the evidence for models of care.

### Value for money

Tamara Chikhradze et al. (709–716) indicate how to improve governments’ purchasing of services.

### From survive to thrive 

Tracey Smythe et al. (717–725) examine the needs of children with congenital conditions.

### Rehab before and after surgery

Cornelia Anne Barth et al. (726–732) describe strategies to improve outcomes in low- and middle-income countries. 

### Planning for the inevitable 

Pete Skelton et al. (744–746) look at what’s required to improve emergency preparedness.

### Audiologists, occupational therapists, physiotherapists, rehabilitation specialists, prosthetists, orthotists and logotherapists

Jim Campbell & Jody-Anne Mills (747–748) outline workforce-related health systems and policy research. 

